# Teaching comprehension of double‐meaning jokes to young children

**DOI:** 10.1002/jaba.838

**Published:** 2021-04-12

**Authors:** Marianne L. Jackson, Rocio M. Nuñez, Dana Maraach, Chelsea J. Wilhite, Jp D. Moschella

**Affiliations:** ^1^ California State University, Fresno; ^2^ University of Nevada, Reno

**Keywords:** humor comprehension, multiple exemplar training, visual prompts

## Abstract

Various forms of humor are an important aspect of social interactions, even at an early age. Humor comprehension is a repertoire that is said to emerge between the ages of 7 and 11 years, and this is primarily attributed to a child's level of cognitive development. The behavioral literature has suggested that various forms of complex verbal behavior, including the use and comprehension of humor, are learned operants that can be taught using systematic teaching procedures. The current study used multiple exemplar training and a three‐step error correction procedure to teach comprehension of double‐meaning jokes to 4 children (2 females and 2 males) aged between 5 and 6.5 years old. All participants demonstrated humor comprehension and appreciation, across multiple exemplars, following training, and maintained this at follow‐up. Implications for use with clinical populations are discussed.

Nonliteral language, the use of words in an abstract rather than literal sense, is a form of complex verbal behavior known to play an important role in social interactions. Common types include metaphors, analogies, sarcasm, and humor. Responding to nonliteral language is essential from a young age (Lazar et al., 1989; Levey & Polirstok, 2011, p. 46), and difficulties in this area can result in issues such as bullying and deficits in social and emotional development (Emerich et al., 2003). Research has shown that forms such as humor can contribute significantly to an individual's overall mental health and physical well‐being (Bennett et al., 2003; Epstein & Joker, 2007; Martineau, 1972; McGhee, 2010; Yoshikawa et al., 2018).

Despite the many documented benefits of humor and laughter, it is a widely overlooked topic in psychology. Much of the work has been conceptual or has focused on the benefits associated with various measures of humor (Epstein & Joker, 2007). A small body of research has examined the development of children's humor and viewed it as a two‐stage process (Aykan & Nalçaci, 2018; McGhee, 1971, 1979, 2002). The first stage, humor comprehension, is described as an understanding of the context of the joke and identification and resolution of the incongruity (the part that doesn't initially make sense). The second stage, humor appreciation, is the overt or covert humor response (e.g., laugh, smile, or positive feeling) as a result of the resolution of the incongruity.

McGhee (1971) examined humor comprehension and appreciation in children aged 5, 7, and 9. He asked participants to state the humorous aspects of a statement, resolve the incongruency, and rate the degree of funniness on a Likert‐type scale. The results suggested that comprehension of such riddles and jokes increased with age, but appreciation may be specific to a particular age range. McGhee (2002) later proposed six increasingly complex stages of humor development, with the final stage including these types of riddles and jokes. Although useful, this description did not identify the specific skills involved or directly address ways to remedy deficits in humor comprehension. Given the social importance of humor, this seems a worthwhile target for behavior‐analytic intervention.

Skinner (1957) laid the groundwork for a conceptual analysis of humor by focusing on the variables that influenced the behavior of the speaker as the joke teller, and that led to a humor response on the part of the listener. He also noted that these humor responses, although often seen as subjective in nature, could also be measured objectively (p. 285).

Skinner (1957, p. 285) pointed out that a verbal response may be funny for several reasons, including that it describes an amusing event or that it is awkward, unexpected, or weak in terms of the sources of control. Much of the subsequent work focused on various accounts of the weak sources of control and the unexpected outcome, drawing on the role of convergent or divergent multiple control (variables coming together to produce a particular response or a given variable affecting multiple responses), supplementary sources of stimulation, thematic variables, and audience control (Epstein & Joker, 2007; Michael et al., 2011; Stewart et al., 2001).

All of these accounts have, with differing terminology, described the initial set‐up of a joke provided by a speaker as serving some type of evocative function for the listener. For example, in the joke set‐up “What do you call a bear with no teeth,” Epstein and Joker's (2007) account suggests that this serves as a type of motivating operation that strengthens certain covert verbal or perceptual responses (e.g., imagining a grizzly bear without teeth or the words toothless or gums). Michael et al. (2011) suggests that it contains thematic variables related to the critical response, which is the element of the joke that will come to be affected by multiple sources of control for the listener, and this will have a practical effect on the listener's response. Stewart et al.’s (2001) account adds that the set‐up of a joke initially forms a complete and coherent relational network that would evoke particular responses on the part of the listener (again, relating to the features of a bear with no teeth).

The other common feature of these behavior analytic accounts is that the set‐up seems insufficient to evoke any particular or meaningful response on the part of the listener, but that supplemental stimulation is required. The punchline provides that stimulation. In our example, “What do you call a bear with no teeth,” the punchline “A gummy bear” would provide supplementary stimulation that, according to Epstein and Joker (2007), would further strengthen the two meanings of the term “gummy” as having no teeth and as a type of candy. For Michael et al. (2011), “gummy” would be the critical response as it is the source of multiple control, and it is thematically related to having no teeth (from the set‐up of the joke). The competing, often covert, responses of the listener to the various sources of control result in a humorous effect. From the perspective of Stewart et al. (2001), the additional stimulation provided by the punchline causes the complete, meaningful, and coherent relational network of the set‐up to collapse initially into incoherence. New relations and networks that presumably include all new forms of multiple control are formed in an unexpected and humorous way. Finally, the extent of the humor response, or how funny a joke is to the listener, depends primarily on the speaker and listener's shared history with all relevant forms of control (Skinner, 1957, p. 240). Michael et al. add that the degree of humor depends on the contextual fit of the additional source of stimulation. If the secondary source of control is introduced only in an attempt at humor, it is unlikely to be funny, but if the secondary source of control is strong in the context and does not exert control over the listener's response until the punchline, it is likely to be regarded as funnier. Epstein and Joker elaborate further on the temporal aspect of the supplementary stimulation provided by the speaker in the punchline and suggest that multiple sources affecting the listener too early during the set‐up or too long after the punchline do not evoke a humor response. That is, if the multiple meanings or sources of strength in the critical response are too obvious to the listener before the punchline is delivered, it fails to be funny. Similarly, if the multiple meanings or sources of strength are not evoked sufficiently by the punchline and need further explanation or the latency to their effect is too long, it fails to be funny. In addition, Dymond and Ferguson (2007) demonstrated the transfer of humorous functions across equivalence classes, suggesting that stimuli can acquire *derived* functions that may set the occasion for a humor response in the presence of relevant stimuli.

Overall, these behavior analytic accounts seem to agree that a joke's set‐up can serve as a motivating operation, discriminative stimulus, and contextual cue to evoke the relevant listener responses to the multiple control and thematic relations established by the supplementary stimulation of the punchline. Given a shared history between the speaker and listener and appropriate timing, the listener is likely to laugh at the joke, often reinforcing the behavior of the speaker telling the joke.

Despite these conceptual accounts, behavior analysis has made little empirical progress in the study of humor. There have, however, been a small number of studies demonstrating successful behavioral interventions for other forms of nonliteral language. The interventions were composed of multiple exemplar training, leading questions, and visual aids to teach children with Autism Spectrum Disorder (ASD) to understand metaphors (Persicke et al., 2012), to detect and respond to sarcasm (Persicke et al., 2013), and to identify deceptive statements (Ranick et al., 2013). These studies suggest that a similar intervention may be used to teach children to understand simple forms of humor, given the common role of multiple and thematic control in the conceptual analyses of these repertoires. Given the social importance and widespread health benefits of humor, such a procedure may be useful for teaching humor comprehension to individuals who have difficulty understanding this form of nonliteral language, such as those with ASD (Emerich et al., 2003; Samson & Hegenloh, 2010). To take a systematic approach, it may be beneficial to evaluate the effectiveness of such an intervention to improve both humor comprehension and appreciation in neurotypical children before evaluating this with children on the autism spectrum.

The purpose of the current study was to assess the effectiveness of an intervention to teach comprehension of double‐meaning jokes to neurotypical children who did not yet demonstrate this skill. We also assessed humor appreciation throughout the study to evaluate the effect of comprehension on the perceived funniness of the joke.

## Method

### Participants and Setting

Four neurotypical children participated in this study. Specific information on each participant's age, setting, and relation to the peer confederate is provided in Table 1. All participants were between 5 and 7 years old. This age range was selected because prior research suggests that this represents the *pre‐riddle* stage of humor development (McGhee, 2002), meaning they would not yet fully understand double‐meaning humor. By parent report, participants had no language, communication, or social delays or disorders and had no history of disruptive behavior that would interfere with experimental sessions. Parents of all participants completed informed consent, and participants agreed to an assent statement that was read aloud to them before the start of the study.

**Table 1:** Participant Demographics

Participant	Gender	Age	Setting	Confederate
Phil	Male	5 years, 1 month	Home	Sibling
Mitch	Male	5 years, 3 months	Preschool	Novel
Gloria	Female	6 years, 5 months	Home	Sibling
Hailey	Female	5 years, 4 months	Preschool	Novel

In addition, three neurotypical children between the ages of 7 and 11 years old participated in the study as peer confederates. Two of the participants, Gloria and Phil, were familiar with their confederates, whereas two of the participants, Mitch and Hailey, were not. Peer confederates spoke in full sentences composed of five or more words and were able to follow directions provided by the experimenters throughout sessions. Parents of peer confederates provided informed consent, and peer confederates stated that they understood and agreed to an assent statement that the experimenter read to them before beginning the study. For all participants and peer confederates, participation was voluntary, and they could withdraw from the study at any time.

Assessments and experimental sessions occurred in the home setting for two participants and a preschool classroom for the other two participants. In each environment, experimenters minimized distractions as much as possible. Session duration was between 20 to 30 min, with breaks available as needed, and participants required between 15 and 22 sessions to complete all phases of the study.

### Materials

Experimenters compiled 76 double‐meaning exemplars (jokes) and 76 nonexemplars (literal statements) from online sources (Kid Jokes, n.d.; Ward, 2018). Nonexemplars were included to examine differential responding (i.e., participants did not just learn to laugh and report that everything was funny). Drawings were created to depict the double meaning of each joke, and these were used as the visual aid in the last of a three‐step prompting procedure. A content knowledge assessment was conducted before the start of baseline sessions to ensure that participants were familiar with both meanings of the target in each joke. Throughout the study, each participant was only exposed to jokes for which they demonstrated the necessary content knowledge. Table 2 provides examples of double‐meaning jokes, the corresponding content knowledge questions, the three levels of prompts, and nonexemplars. Each participant was exposed to a mean of 51.2 jokes (range 48‐57) throughout the study. All jokes were novel in baseline and were not repeated within this phase, although these jokes could be used again during intervention and follow‐up if needed. During intervention and follow‐up, jokes were only repeated if experimenters had used all the jokes allocated for these phases. No joke was used more than three times with a mean use of 2.1 times throughout the study. Twenty‐five novel jokes were retained for each participant's postprobe sessions to ensure sufficient novelty, and these were randomly selected by the experimenter. Nonexemplars were arranged in the same manner.

**Table 2:** Sample Exemplars, Content Knowledge Questions, Prompts, and a Nonexemplar

Exemplars and Nonexemplars	Content Knowledge Questions	Leading Question	Specific Question	Visual Aid
Q: Why was 6 afraid of 7? A: Because 7 ate 9	What number comes after 7 and before 9? What does it mean to say that I ate chips?	What are two meanings of the word ate/eight*?	What number comes after 7 and before 9? What does it mean to say “yesterday I ate” something?	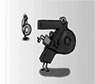
Q: What do you call a bear with no teeth? A: A gummy bear!	What kind of candy is a gummy? What is the pink part of your mouth that your teeth grow out of?	What are two different meanings of gummy?	What might you say about something with no teeth? What's a type of candy bear you eat?	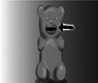
Q: What sound does a dog make? A: Woof woof	N/A	N/A	N/A	N/A

^*^ didn't say both as they are homonyms.

### Dependent Variables and Interobserver Agreement

The primary dependent variable in this study was humor comprehension, and humor appreciation was measured as a secondary dependent variable. Humor comprehension was measured using a four‐point system, similar to the one used by McGhee (1971). For exemplars, a score of 4 was given for responses that identified the word that composed the double meaning, stated the two meanings, and explicitly related them. A score of 3 was given for responses that identified the word that composed the double meaning and stated the two meanings but did not explicitly state how the words were related. A score of 2 was given for responses that only identify one meaning of the double meaning word. Lastly, a score of 1 was given for responses that repeated the joke, did not identify any targets of the incoherency or double meaning, or for no relevant response.

For nonexemplars, a score of 4 was given for responses that identified the nonexemplars as not funny and provided a correct explanation that included both points in the literal statement and stated it made sense. A score of 3 was given for responses that identified nonexemplars as not funny but did not provide a correct explanation or provided an explanation that only referenced one point of the literal statement. A score of 2 was given for responses that identified nonexemplars as funny and offered no further explanation that referenced the targets in the literal statement. Lastly, a score of 1 was given for responses that identified the nonexemplars as funny and attempted an explanation of why it was funny that included the points of the literal statement.

**Figure 1:**
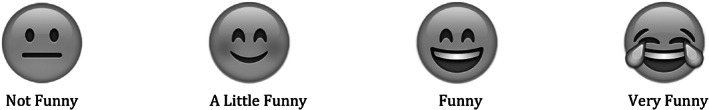
Rating Scale with Emojis Representing Differing Levels of Humor Appreciation *Note*. These were scored from 0 (not funny) to 3 (very funny).

Humor appreciation was assessed by the participants' latency to smile or laugh and by the score they indicated on a Likert‐type emoji scale. A smile was defined as an upward curvature of the edges of the lips, with or without the display of teeth, and without vocal sound. A laugh was defined similarly but with a repetitive vocal sound. A timer was started at the end of the delivery of the exemplar or nonexemplar and stopped when the participants displayed a smile or laughed or after 10 s had elapsed. A Likert‐type emoji scale was used to measure participant reports of the extent to which they found a statement to be funny (Figure 1). Each emoji was accompanied by a textual description of *not funny, a little funny, funny*, or *very funny* and assigned a number for data collection purposes (ranging from 0 for not funny to 3 for very funny). Nonexemplars were included to ensure that participants were differentially responding to jokes and nonjokes and not learning to laugh at any statements presented by the researcher. In typical humor responses, a short latency to smile or laugh often correlates with jokes rated as funny, and a longer latency or no smile or laugh often correlates with ratings of not funny or less funny (Cunningham & Derks, 2005; Mireault et al., 2015).

Interobserver agreement (IOA) data were collected for 46% of trials, across all participants and phases. Agreement was calculated by comparing the data of the primary data collector with that of a second data collector on a trial‐by‐trial basis and dividing the number of total agreements by the number of agreements plus disagreements, multiplied by 100. For latency measures, responses were marked as agreements if they were within 0.3 s of each other. Across participants, mean IOA was 99.4% (range: 98.7%‐100%) for the comprehension measure, 95.4% (range: 91.8%‐ 98.3%) for latency to smile or laugh, and 100% for the funniness rating scale.

### Independent Variable and Integrity Measures

The independent variable in this study was multiple exemplar training with a three‐step prompting hierarchy composed of a leading question, a specific question, and a visual aid (see Table 2 for examples). This intervention was selected as it had been successful in teaching other forms of nonliteral language (e.g., Persicke et al., 2012). Experimenters delivered verbal praise immediately following the correct identification of the double meaning within a joke and initiated the prompting hierarchy following an incorrect response. This began with a leading question and progressed as needed until a correct response was evoked. If none of the prompts were successful, a full model of the correct response was provided, and participants were asked to imitate the response. No differential consequences were provided for appreciation measures (latency to smile/laugh or funniness ratings).

#### Experimenter Training and Procedural Integrity

All experimenters and data collectors were trained using a behavioral skills training package (BST; Miltenberger, 2012, pp. 217‐234) until they reached 90% accuracy across two consecutive sessions. Experimenters were provided with a checklist of implementation steps to reference when needed during sessions and a written list of exemplars and nonexemplars for each session. The same checklist was used for data collection on procedural integrity, with 26 items that included the ordering and presentation of exemplars and nonexemplars, use of timers, rating scales, and scoring rubrics, delivery of programmed reinforcers, and implementation of the three‐step prompting hierarchy. The mean procedural integrity across participants and phases was 98.9% (range 90‐100%). One noticeable failure of procedural integrity occurred for Phil during his first posttraining probes when the experimenter delivered four nonexemplars instead of five, making his possible score out of 16 and not 20.

#### Peer Confederate Training

Peer confederates were trained to deliver exemplars and nonexemplars using BST, and their training focused on accurately repeating the joke or literal statement provided, pausing briefly before the punchline or answer to the question, and using a varied intonation in delivering the punchline of exemplars or a lack of varied intonation for nonexemplars. Training continued until peers delivered exemplars and nonexemplars at 90% accuracy across two practice sessions. In addition, peers practiced each joke and literal statement with the experimenter prior to each session and were provided with feedback and another opportunity to rehearse if needed. During initial training and presession rehearsals, correct aspects of delivery were praised, and feedback was provided as needed for missing or incorrect components.

### Experimental Design

A nonconcurrent multiple‐baseline‐design across participants was used to assess the effects of multiple exemplar training on humor comprehension. The intervention was implemented after three to seven baseline sessions and subsequent phases implemented based on performance on humor comprehension. Posttraining phases assessed generalization to novel exemplars and nonexemplars in the absence of programmed reinforcement, and follow‐up probes evaluated the extent to which this maintained over time. For both humor appreciation measures (latency and rating), data were also examined for the degree of differentiation in responding to exemplars and nonexemplars across all baseline, posttraining, and follow‐up probes.

### Procedures

All assessments and intervention sessions began with 10 min of a preferred activity with the experimenter or peer. No instructions were given, and the experimenter or peer interacted freely throughout this time. Participants were informed that the activity was over, assisted in clearing away any materials, and directed to sit at the table with the experimenter or peer confederate. Peers provided the exemplars and nonexemplars during all baseline, postprobe, and follow‐up sessions. The experimenters implemented all procedures in the intervention sessions.

#### Assessments

At the beginning of the study, all participants were asked about their favorite toys, characters, movies, etc. Their answers were used to identify tangible items that would be preferred and may function as reinforcers for appropriate session behaviors. These included small toys, stickers, and books that were placed in the prize box and delivered at the end of each session, regardless of the accuracy of participants' responses.

Participants completed a content knowledge assessment prior to any baseline sessions. This assessment was designed by the experimenters to assess participants' knowledge of the various meanings of the homonym in each double‐meaning joke (see Table 2 for examples). Questions were semirandomly ordered to ensure that content questions for the same joke did not appear together. Throughout the study, participants were only exposed to exemplars for which they had demonstrated the necessary content knowledge (i.e., they could respond to all relevant meanings of the homonym in the exemplar). The assessment was conducted individually and included approximately 165 questions, with two to three questions per joke. Participants had 5 s to respond to each question, and all responses were followed by brief, nonspecific praise (e.g., “thanks,” “ok,” “you're sitting so well”), regardless of accuracy. Sessions did not last longer than 30 min and breaks were interspersed as needed.

#### General Session Procedures

All participants and peers sat at a table, and the experimenter said, “You are going to hear things that are funny because they are jokes, and things that are not funny because they are not jokes.” This was followed by instructions on what a joke was and what it was not and interspersed with questions about what was said to ensure that children were attending to the instruction and could repeat it. These were as follows:

A joke usually has one word that can mean two different things. How many things can one word mean? When one word means two different things, that sometimes makes a joke funny. What makes a joke funny? Something is not a joke when what you say only means one thing, and it makes sense. When is something not a joke?

Prior to all sessions, the experimenter quasirandomly ordered five exemplars and five nonexemplars, with no more than two of the same type occurring consecutively. After the experimenter or peer confederate had delivered the punchline or literal statement, the experimenter started a timer to measure latency to smile or laugh. After the participant smiled or laughed, or 10 s had elapsed, the experimenter presented the emoji scale (Figure 1) and asked the participant to rate how funny it was. The experimenter then asked the participant why it was funny or not funny (based on their previous response), waited 5 s for the participant to initiate a response, and scored this on the respective scale for comprehension. Across five exemplars and five nonexemplars, this allowed for a total comprehension score of 20 for each session. A mastery criterion for humor comprehension was set at 15 out of 20 over three consecutive sessions for exemplars and nonexemplars. For exemplars, a score of 15 or greater indicated that participants had identified the double meaning within the joke on most trials and may also have explicitly related the two. Scores of 14 or below indicated that, on some trials, participants failed to identify the two meanings or relate them. For nonexemplars, a score of 15 or above indicated that, on most trials, participants could identify that the literal statements were not jokes and could explain why.

#### Joke Telling Probes

Although joke telling was not directly taught in the intervention, it was assessed to see if the comprehension intervention produced any changes in joke‐telling. During the first session of baseline and posttraining phases, the peer confederates asked the participant, “Can you tell me a funny joke?” Participants were given 10 s to initiate a response and had three opportunities throughout the session. All responses were recorded verbatim. Data were also collected on the peer's latency to smile or laugh and the peer's rating of funniness on the emoji scale. Peers could respond freely, and the experimenter provided no feedback during these probes.

#### Baseline/Posttraining Probe Sessions

All subsequent baseline and posttraining probe sessions began with the description of exemplars and nonexemplars, and the peer then delivered five novel exemplars and five novel nonexemplars in a semirandomized order, using the written reminders as needed. Appreciation and comprehension measures were taken after each presentation, and then the next trial began. The experimenter did not provide feedback during any of the baseline or posttraining probe sessions. All exemplars and nonexemplars used during baseline and posttraining probe sessions were novel to the participant, and no repetitions were used.

#### Intervention (Multiple Exemplar Training)

Sessions were conducted similarly to the probe sessions described above, with the exception that specific praise followed correct responses, and a three‐step prompt hierarchy followed incorrect responses. Following the delivery of an exemplar, appreciation measures were collected. The experimenter then asked the comprehension question, “tell me why it's funny” or “tell me why it's not funny” if they had indicated that it was not. A correct response that stated both meanings of the homonym in the joke and explicitly related them was followed by verbal praise, and the trial ended. Responses that did not identify both meanings or did not explicitly relate the two meanings were followed by one of three increasing prompt levels, and the exemplar was re‐presented at each level to allow the participant to respond independently. If they did not respond independently, the next level of the prompt hierarchy was presented. Prompting on any given trial began with a leading question, followed by a specific question, and then a visual prompt (examples can be seen in Table 2). If the participant still did not respond correctly after the visual prompt, the experimenter provided the correct answer and used the visual depiction to explain the double‐meaning and why it was funny.

During comprehension measures for nonexemplars, correct responses identifying that the statement was not a joke were followed by praise. Incorrect responses that attempted to identify a humorous double‐meaning were followed by corrective feedback, informing participants that the statements were not jokes, and the next trial began. At the end of each session, participants were praised for completing the session and provided with the opportunity to pick an item from the prize box.

Once participants met the mastery criterion of 15 out of 20 across a minimum of three consecutive sessions for exemplars and nonexemplars, they moved on to posttraining probes. These were conducted as described and only included novel jokes to assess generalization across exemplars in the absence of programmed reinforcement (praise).

#### Follow‐up Sessions

Follow‐up sessions were conducted 2 weeks after the last posttraining probe session to assess the maintenance of any intervention effects. They were conducted similarly to baseline and posttraining sessions with the exception that both novel and previously used exemplars and nonexemplars were included, and participants were not asked to tell jokes. If participants failed to maintain a score of 15 out of 20 during the maintenance session, intervention sessions were reimplemented until they met the mastery criterion again, and then a second postprobe or maintenance check was conducted.

## Results

### Comprehension Measures

**Figure 2:**
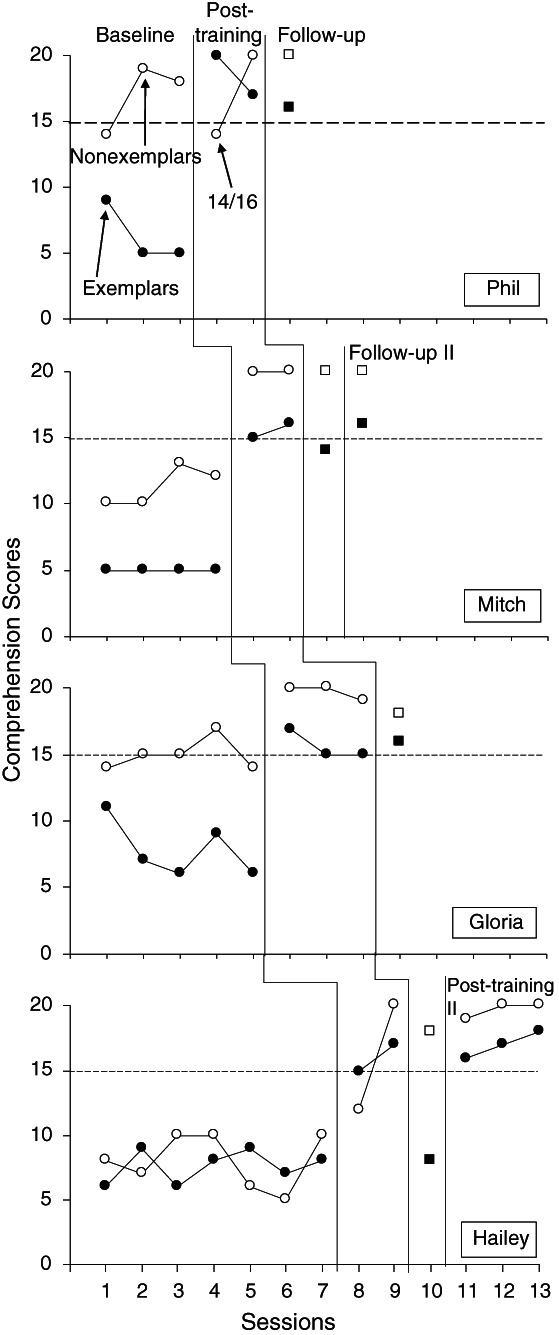
Comprehension Measures Across Baseline, Posttraining, and Follow‐up Sessions *Note*. Follow‐up probes contained some repetition of exemplars (closed) and nonexemplars (open) and are denoted by squares. The dashed line indicates the mastery level.

Figure 2 shows the humor comprehension scores for all participants across baseline, posttraining, and follow‐up phases. All participants showed relatively low and stable scores during baseline, scoring less than 10 points across five exemplars (four points available for each), meaning that none of the participants was consistently able to identify the double meaning or explicitly relate the two meanings across exemplars. Participants either stated one meaning of the double meaning word but did not identify the second meaning or source of humor (scoring 2 for that exemplar) or gave some other response that referenced or repeated the joke but was irrelevant to the source of humor (scoring 1 for that exemplar). Two participants, Phil and Gloria, scored relatively high on the nonexemplars in baseline, with scores ranging from 14 to 18 out of 20, indicating that they were able to identify many of the nonexemplars as not funny. The other two participants, Mitch and Hailey, scored lower in baseline for nonexemplars, with scores ranging from 5 to 13, suggesting that they were only able to identify a few nonexemplars as not funny.

Following multiple exemplar training with experimenters, all participants met the mastery criterion of 15 or greater out of 20 points across at least three consecutive sessions on novel exemplars and nonexemplars. The number of intervention sessions required for each participant to meet mastery is shown in Table 3. Overall, this required a mean of 9.75 sessions (range, 6 to 13), and all participants continued to meet this criterion during posttraining probes with novel exemplars and nonexemplars, showing generalization to new stimuli and to peers. When assessed for maintenance 2 weeks later, two of the participants, Phil and Gloria, continued to demonstrate mastery, whereas the other two participants, Mitch and Hailey, required a second intervention phase with four and seven sessions, respectively. It should be noted that Mitch and Hailey were exposed to an extended number of sessions during the second intervention phase to increase the likelihood of maintenance but met the initial mastery criterion with three and five sessions, respectively. Mitch completed a final posttraining probe and maintenance check one month later and demonstrated mastery. Hailey completed three posttraining probe sessions without a maintenance session as she moved out of state and was not available for follow‐up sessions. She met the mastery criterion on all posttraining probe sessions.

**Table 3:** Number of Multiple Exemplar Training (MET) Sessions to Meet and Maintain Mastery

Participant	MET	MET II
Phil	9	N/A
Mitch	11	4
Gloria	13	N/A
Hailey	6	7

### Appreciation Measures

**Figure 3:**
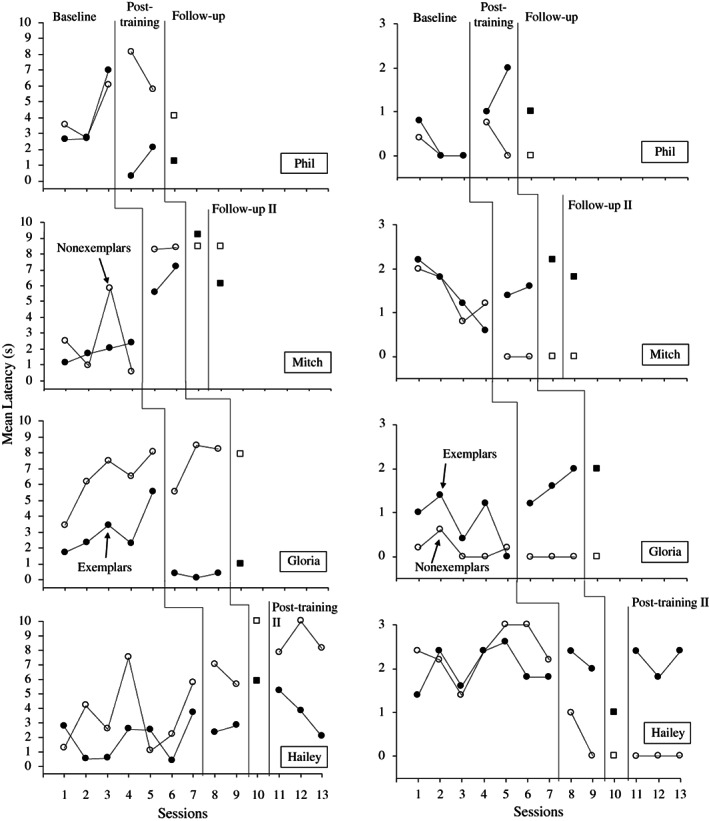
Appreciation Measures Across Baseline, Posttraining, and Follow‐up Sessions *Note*. Follow‐up probes contained some repetition of exemplars (closed) and nonexemplars (open) and are denoted by squares. Ratings of funniness (right) were scored from 0 (not funny) to 3 (very funny).

Figure 3 shows the humor appreciation data for latency to smile or laugh and the ratings of funniness. Differentiation between the data for exemplars and nonexemplars, with shorter latencies and higher ratings for exemplars than nonexemplars, would indicate that participants found exemplars funnier than nonexemplars. In baseline, all participants showed either overlapping and undifferentiated latencies across exemplars and nonexemplars or increasing latencies for exemplars. Gloria did show differentiated responding with shorter latencies to smile or laugh for exemplars than nonexemplars but with an increasing trend on both. Phil, Mitch, and Hailey all had overlapping baselines on this measure. Correspondingly, all participants showed overlapping ratings of funniness across exemplars and nonexemplars, except for Gloria, who showed some differentiation with exemplars rated as slightly funnier than nonexemplars, but with a decreasing trend.

Once participants had met the mastery criterion for comprehension (the primary dependent variable), they also showed differentiated responding to exemplars and nonexemplars on both appreciation measures. More specifically, all participants demonstrated shorter latencies to laugh or smile for exemplars than nonexemplars and higher ratings of funniness for exemplars than nonexemplars; however, there were some notable variations across phases and participants, and these tended to vary with the level of comprehension, particularly for sessions in which participants' responding dropped below the mastery criterion (session 7 for Mitch and session 10 for Hailey).

### Joke‐Telling Probes

During initial baseline probes and posttraining probes, peer confederates asked participants to tell them a joke. They were given three opportunities to do so, and these were scored using the peer's rating of funniness and latency to smile or laugh to allow for some degree of age‐appropriate humor (i.e., what the adult experimenter finds funny may be different from what the similarly aged peer finds funny). Joke‐telling was not directly taught at any time during the intervention. Two participants showed improvements in joke‐telling, one appeared to worsen, and one stayed the same. More specifically, Gloria increased from one to three jokes rated as funny and Hailey from no jokes to one joke rated as funny by peers. Phil told three jokes rated as funny in baseline sessions and one joke that was not rated as funny in the posttraining session. Mitch did not attempt to tell any jokes in baseline or posttraining sessions. Anecdotal reports from parents and participants suggest that some successful jokes were memorized and recalled accurately, although the degree of comprehension was not directly assessed. Overall, it seems that the intervention to teach humor comprehension did not result in a consistent improvement in joke‐telling skills.

### Social Validity

Parents of 3 out of 4 of the participants completed a social validity survey at the start and end of the study. Hailey's parent was unavailable to complete the social validity survey at the end. At the beginning and end of the study, all parents *agreed* or *strongly agreed* that humor was a common and important part of children's interactions and that it could facilitate social relationships. At the end of the study, they also agreed or strongly agreed that their child enjoyed being a part of the study, that they saw changes in their child's understanding and appreciation of humor, that these procedures should be used to teach other forms of humor and nonliteral language, and that they were happy their child participated in the study.

Participants also completed a social validity survey that was read aloud to them before and after the study (again, Hailey was unavailable for the survey at the end of the study). At the start of the study, three participants reported that jokes were an important part of being with friends and that they understood jokes. At the end of the study, participants said that they enjoyed being part of the study and that they understood the jokes better by the end of the study.

## Discussion

All participants demonstrated humor comprehension for double‐meaning jokes, following the intervention, and generally showed humor appreciation measures in line with their comprehension. While all participants demonstrated mastery with novel exemplars in the posttraining probe sessions, two of the participants (Mitch and Hailey) dropped below the mastery criterion in follow‐up and required an additional intervention phase. Three of the four participants then demonstrated continued comprehension and appreciation (the fourth participant was unavailable for follow‐up).

Although humor comprehension was the main focus of the intervention, measures of humor appreciation were also important. More specifically, this addressed the concern that explicit teaching of humor comprehension may detract from the humorous effect itself (i.e., it is a common experience that a joke that requires a detailed explanation, although understood, is not very funny). Although all participants showed greater differentiation in appreciation measures after meeting the mastery criterion for humor comprehension, there were variations across participants that are worthy of further discussion.

During intervention and follow‐up sessions, Phil often rated repeated exemplars as less funny and showed longer latencies to laugh or smile. During these trials he even told the experimenter, “I've already heard that one.” Mitch generally demonstrated longer latencies to smile and laugh than other participants. This often occurred because he responded to comprehension questions immediately after the punchline of the joke, even though these questions were not asked until the 10‐s interval had elapsed, and then he smiled or laughed. Both Mitch and Hailey demonstrated longer latencies to smile or laugh during the first follow‐up session when they both dropped below the mastery criterion for humor comprehension. Hailey also provided lower ratings of funniness for exemplars during this session. This is interesting and seems to be consistent with the premise that humor comprehension is an important component of humor appreciation. Overall, Gloria showed clear and consistent patterns of differential responding in her appreciation measures following the intervention.

These idiosyncratic aspects of humor appreciation suggest that several variables may impact these measures. Appreciation may have been amenable to social contingencies, and future research could investigate alternative measures of humor appreciation that differ across humor types and contexts. Another issue that seemed to affect appreciation was the repetition of jokes during intervention and follow‐up sessions. This was done in an *as‐needed* manner, and future studies may want to more systematically evaluate the effects of repeated presentations of exemplars.

There was also some variation in responses to joke‐telling probes across participants. Although joke‐telling was not directly taught during the study, three of the participants did attempt to tell jokes in baseline and postraining sessions and many of them were successful (i.e., their peer rated the jokes as funny). Anecdotal reports suggest that they had memorized and practiced these jokes prior to the sessions. Although most people asked to tell a joke would retell a joke they had been told by someone else previously, humor comprehension measures taken in baseline and errors made in delivery suggest that either the participants did not understand why the jokes they told were funny or that they understood these specific exemplars but could not apply this to novel exemplars. Future studies could ask participants why the jokes they told were funny, and this may provide greater insight into the degree of comprehension of jokes told by participants during baseline and posttraining sessions. It may also be helpful to investigate ways to promote the emergence of joke‐telling from humor comprehension or vice versa, perhaps by using more exemplars of joke‐telling throughout the study or by teaching both in an interspersed format, similar to multiple exemplar instruction (LaFrance & Tarbox, 2019).

It is also worth noting that the study was conducted in a preschool setting for Mitch and Hailey, who both required a second intervention phase, and in the home setting for Phil and Gloria, who showed generalization and maintenance after the first intervention phase. Moreover, Phil and Gloria's confederate peer was their older sibling. Anecdotal reports suggest that parents and siblings of the home‐based participants shared more jokes with the participants throughout the study and beyond. This may have enhanced the effectiveness of the intervention and maintenance of the effects.

There are several limitations to this study that warrant further discussion. The design of this study was a *nonconcurrent* multiple‐baseline design, and the intervention was staggered across participants based on the number of baseline points and stability, not the performance of the previous participant. Future studies may further strengthen the demonstration of experimental control by considering these issues.

It may also be necessary to refine some aspects of the methodology. For example, the criterion for mastery was two consecutive sessions with a minimum score of 15 out of 20, meaning it was possible to meet this without identifying the double meaning of some exemplars (e.g., scoring 4, 4, 4, 1, 2 in which the double meaning is not identified for the last two exemplars). Although a review of the data suggests this was not a substantial problem for participants, future studies may wish to use a criterion that requires a score of 3 or more on all exemplars and nonexemplars. In addition, the rule statement given to participants described a joke as funny when one word has two meanings, and stated that something is not a joke when it only means one thing, and it makes sense. This is a simplified description of a joke and specifically applies to the type of jokes used in this study. Furthermore, the intervention teaches participants to *make sense* of the joke, so this may need to be refined in future studies.

A further limitation of this study is that no measure was taken of humor comprehension or joke‐telling in a more relaxed and typical social environment. The inclusion of playtime at the start of each session and peers in the baseline, posttraining, and follow‐up sessions, were intended to reduce possible reactivity. However, the situation itself was still quite contrived. In future studies, it may be useful to include other age‐appropriate, fun activities that promote laughter and silliness with peers and maybe even include caregivers or other familiar adults in place of the experimenter. In addition, there were no measures of humor comprehension over more extended periods, nor were there measures of participant preference for the exemplars or the peers who delivered them. These measures may be important to the broader implications of such an intervention. Future research may be further informed by a descriptive study that provides some normative data on the occurrence of double‐meaning jokes among relevant age‐groups.

This study was conducted with children who were younger than the age at which comprehension of double‐meaning jokes should emerge, according to the existing literature. This intervention may be useful for teaching humor comprehension to individuals who have consistent difficulty with humor comprehension, specifically with double‐meaning jokes. For example, research has suggested that some individuals with ASD may exhibit lower levels of humor comprehension than their neurotypical peers (e.g., Emerich et al., 2003; Samson & Hegenloh, 2010), and this may be specific to verbally complex types of humor such as jokes and riddles (Reddy et al., 2002). Implementation of the intervention for this purpose may require some modification. More contrived forms of reinforcement may be needed on a denser schedule, and it may be necessary to assess prerequisite skills, such as perspective‐taking and multiple control (Reddy et al., 2002; Rehfeldt et al., 2007; Sundberg & Sundberg, 2011). It is also important to note that the prompting hierarchy often took some time, and participants were sometimes distracted before the end of a fully prompted trial. It may be fruitful to look for ways to reduce the extent of this while maintaining its effectiveness.

The visual images used in the prompt hierarchy were time‐consuming to create and required some creative expertise. In addition, one participant repeatedly asked to see the images, and as the most intrusive level of prompt, this could lead to a type of prompt dependence (i.e., the image may be more reinforcing than the correct independent response). It may be useful to look for alternative ways to teach children to understand the humorous nature of the double meaning contained within the joke. One option may be to teach children to visualize a humorous combination of the two meanings in a manner similar to Kisamore et al. (2011). Kisamore et al. taught preschool children to use visual imagining to solve categorization problems, and results suggest that this strategy may be most effective when used in combination with rule statements about when to use it. A strategy of visual imagining may not only eliminate the need to create drawings for each exemplar but may also reduce the possibility of prompt dependence while moving closer to a real‐life experience of what happens when we understand double‐meaning jokes.

Finally, a similar protocol may be helpful to teach children to understand other types of jokes (e.g., knock‐knock jokes or riddles). It may allow for a behavior analytic account of both humor development and the broader spectrum of humor in its various forms. Given the overall importance humor is said to have in everyday life and social interactions, this could be a meaningful and fun addition to a behavior analytic account of complex language.
